# DIO-SLAM: A Dynamic RGB-D SLAM Method Combining Instance Segmentation and Optical Flow

**DOI:** 10.3390/s24185929

**Published:** 2024-09-12

**Authors:** Lang He, Shiyun Li, Junting Qiu, Chenhaomin Zhang

**Affiliations:** 1 Faculty of Mechanical and Electrical Engineering, Kunming University of Science and Technology, Kunming 650500, China; 20222203185@stu.kust.edu.cn (L.H.);; 2 College of Mechanical Engineering, Zhejiang University of Technology, Hangzhou 310014, China; qiujt@zjut.edu.cn

**Keywords:** dynamic SLAM, instance segmentation, dense optical flow, dynamic feature point removal, point cloud reconstruction, octree

## Abstract

Feature points from moving objects can negatively impact the accuracy of Visual Simultaneous Localization and Mapping (VSLAM) algorithms, while detection or semantic segmentation-based VSLAM approaches often fail to accurately determine the true motion state of objects. To address this challenge, this paper introduces DIO-SLAM: Dynamic Instance Optical Flow SLAM, a VSLAM system specifically designed for dynamic environments. Initially, the detection thread employs YOLACT (You Only Look At CoefficienTs) to distinguish between rigid and non-rigid objects within the scene. Subsequently, the optical flow thread estimates optical flow and introduces a novel approach to capture the optical flow of moving objects by leveraging optical flow residuals. Following this, an optical flow consistency method is implemented to assess the dynamic nature of rigid object mask regions, classifying them as either moving or stationary rigid objects. To mitigate errors caused by missed detections or motion blur, a motion frame propagation method is employed. Lastly, a dense mapping thread is incorporated to filter out non-rigid objects using semantic information, track the point clouds of rigid objects, reconstruct the static background, and store the resulting map in an octree format. Experimental results demonstrate that the proposed method surpasses current mainstream dynamic VSLAM techniques in both localization accuracy and real-time performance.

## 1. Introduction

Simultaneous Localization and Mapping (SLAM) technology is fundamental to robotic perception, enabling autonomous navigation and exploration of unknown environments. It utilizes sensors such as LiDAR, cameras, and Inertial Measurement Units (IMUs) to perceive unknown environments and progressively construct a globally consistent map that reflects the real-world environment while continuously updating the robot’s current position during dynamic processes [1]. In recent years, with the advancement of computer vision and deep learning technologies, camera-based visual SLAM has become integral to applications like autonomous driving, virtual reality (VR), and augmented reality (AR). Compared to LiDAR-based SLAM technology, VSLAM relies on images as the primary medium of environmental perception, making it more aligned with human understanding [2,3]. Visual sensors encompass a variety of cameras, including monocular, stereo, event-based, and RGB-D (Red–Green–Blue–Depth) cameras. Among these, RGB-D cameras, which utilize depth sensors to capture depth information, are particularly promising due to their ease of configuration, compactness, and cost-effectiveness [4,5].

The mainstream techniques in visual SLAM include the direct method and the feature-based method. The direct method estimates camera motion based on pixel intensity information, eliminating the need for keypoints or descriptors. In contrast, the feature-based method, which dominates VSLAM, extracts feature points from real-time images, establishes an optimization model to estimate the camera pose, and constructs an environmental map by analyzing the relationships between these feature points [6]. Among feature-based RGB-D SLAM algorithms, the ORB-SLAM series is the most representative. Introduced in 2015, ORB-SLAM is one of the most comprehensive and user-friendly SLAM systems, standing as the pinnacle of mainstream feature-based SLAM algorithms. ORB-SLAM2 [7], building on ORB-SLAM [8], introduced a global optimization module, enhancing the system’s robustness, accuracy, and efficiency. ORB-SLAM3 [9] further extended ORB-SLAM2 by adding the multi-map functionality Atlas [10], which allows the system to immediately reconstruct a new sub-map during tracking loss, thus preventing interruptions in map updates. Additionally, ORB-SLAM3 supports the integration of visual and inertial sensors, improving the stability of feature point tracking in low-texture environments.

**Figure 1:**
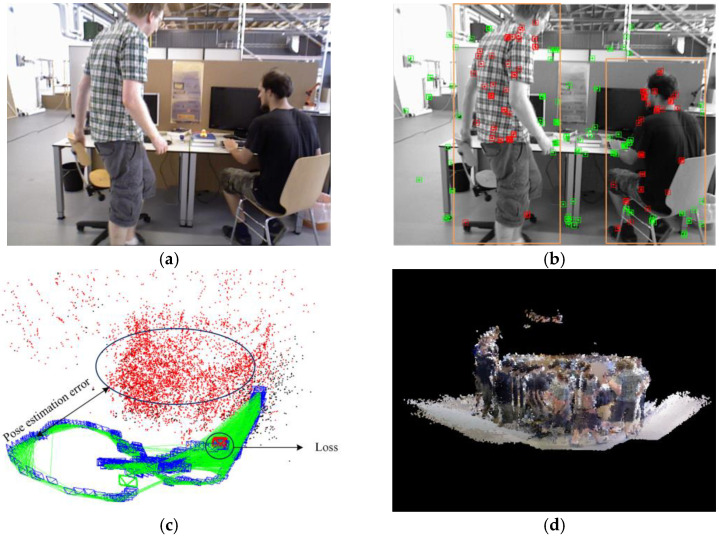
Performance of the traditional ORB-SLAM3 algorithm in highly dynamic environments. (**a**) Image of the highly dynamic scene. (**b**) Feature point extraction in the highly dynamic scene, where yellow boxes indicate moving objects and dynamic feature points are marked in red. (**c**) Comparison between the estimated camera pose and the ground truth camera pose. (**d**) Reconstruction results of dense mapping.

The ORB-SLAM series algorithms discussed above are all based on the assumption of a static environment. However, in the real world, many dynamic objects exist that can cause pose tracking failures or irreversible damage to the map. As illustrated in Figure 1a, a highly dynamic scene is depicted, with two people walking back and forth while dragging a chair. Extracting feature points from Figure 1a results in Figure 1b, where a significant number of dynamic ORB feature points cluster around the moving objects within the yellow box. This incorrect feature extraction leads to pose estimation errors or even failures, as seen in Figure 1c. This issue arises because, although ORB-SLAM3 performs exceptionally well in purely static environments, the algorithm uses feature points from dynamic objects to estimate the camera’s pose when such objects enter the camera’s field of view. Furthermore, during dense point cloud reconstruction, as shown in Figure 1d, the algorithm fails to filter out dynamic point clouds, making it difficult to obtain accurate 3D scene information. Additionally, for manually moved rigid objects in the scene, such as balloons, boxes, and chairs, the traditional ORB-SLAM3 algorithm struggles to effectively track their true motion state. Therefore, developing a VSLAM system capable of efficiently handling dynamic scenes is of great practical significance.

To address the aforementioned issues, this paper introduces DIO-SLAM, which integrates instance segmentation with dense optical flow algorithms via optical flow consistency. This integration effectively distinguishes between non-rigid objects, stationary rigid objects, and moving rigid objects in the scene. Once moving rigid objects become stationary, their static feature points are retained for tracking, optimization, and map construction. The primary innovations of this paper are summarized as follows:

1. To deal with the issue of excessive noise in existing dense optical flow algorithms, which makes it difficult to accurately identify moving objects, this paper proposes an optical flow consistency method based on optical flow residuals. This method effectively removes optical flow noise caused by camera movement, providing a solid foundation for the tight coupling of dense optical flow and instance segmentation algorithms.
2. A motion frame propagation method is proposed, which transfers dynamic information from dynamic frames to subsequent frames and estimates the location of dynamic masks based on the camera’s motion matrix. By compensating for missed detections or blurring caused by significant object or camera movements, this approach reduces the likelihood of detection thread failure, thereby enhancing the accuracy and robustness of the system.

The remainder of this paper is organized as follows. Section 2 provides an overview of related work. Section 3 introduces the overall system framework and working principles of DIO-SLAM. Section 4 details how the detection and optical flow threads are combined to determine the motion state of rigid objects, explaining the principles of optical flow residuals, optical flow consistency, and the motion frame propagation method. It also describes the method for tracking the point clouds of moving rigid objects within the dense mapping thread. Section 5 evaluates the accuracy of DIO-SLAM on relevant datasets, compares camera pose accuracy with state-of-the-art dynamic VSLAM systems, and demonstrates the effectiveness of each module through ablation experiments. The accuracy of the point clouds obtained from dense mapping is also verified. Finally, tests are conducted in real-world scenarios. Section 6 provides a summary of the paper.

## 2. Related Work

### 2.1. Algorithms Based on Geometric Constraints and Detection Segmentation

In recent years, several dynamic VSLAM algorithms have been introduced that use detection or segmentation methods to extract and remove a priori objects. Detect-SLAM, introduced in [11], utilizes SSD [12] (Single Shot Multibox Detector) for object detection only on keyframes, while the remaining frames propagate motion probability frame by frame. However, this method cannot accurately determine the true motion of a priori dynamic objects, potentially leading to the erroneous removal of static feature points. MaskFusion [13] employs Mask R-CNN [14] for instance segmentation of objects in images and combines it with ElasticFusion [15] to track dynamic objects and reconstruct a 3D map. DS-SLAM [16] employs the SegNet [17] deep fully convolutional neural network for semantic segmentation, combined with motion consistency detection to identify dynamic regions, but it can only remove dynamic feature points from human bodies. SOLO-SLAM [18] utilizes the SOLO-V2 [19] instance segmentation algorithm as a replacement for Dyna-SLAM’s Mask R-CNN algorithm, addressing Dyna-SLAM’s real-time performance issues, but it still lacks the capability to accurately determine the true motion state of a priori dynamic objects.

Most of these approaches avoid the impact of moving objects on VSLAM performance by removing a priori feature points, without precisely identifying dynamic regions on objects, and instead directly removing the entire a priori object. To determine the motion state of objects without prior information, several methods based on geometric constraints have been suggested. Dyna-SLAM [20] and its successor, Dyna-SLAM II [21], which enhances multi-object tracking capabilities, accurately remove dynamic feature points by combining Mask R-CNN instance segmentation results with multi-view geometry. Dyna-SLAM also repairs static backgrounds using a missing map completion algorithm, but it suffers from significant time consumption and poor real-time performance. The literature [22] proposes an accurate geometric constraint recognition method based on the invariance of static point positions, which can complement semantic segmentation to jointly eliminate dynamic feature points. MVS-SLAM [23] introduces a self-motion estimation module that enhances both the speed and accuracy of initial camera pose estimation. It also tightly integrates semantic information with multi-view geometry to effectively remove dynamic feature points.

### 2.2. Algorithms Based on Optical Flow and Detection Segmentation

To resolve the aforementioned issues, some approaches have introduced optical flow methods to accurately detect the actual motion state of objects, thereby obtaining more stable feature points for recovering camera poses. FlowFusion [24] compares an optical flow map, generated by offsetting the previous frame based on a fully static assumption, with the actual optical flow obtained through the PWC Net [25] to identify moving objects and remove their point clouds during reconstruction. The combination of deep learning methods with optical flow can effectively locate dynamic objects, offering better resistance to interference from moving objects compared to using optical flow or deep learning alone. Several related works have presented solutions. References [26,27,28] suggest using object detection algorithms in conjunction with the Lucas-Kanade [29] (LK) optical flow algorithm to reject dynamic points in keyframes. Nevertheless, object detection algorithms may not accurately detect objects, potentially leading to the erroneous removal of static feature points. To address this issue, DM-SLAM [30] combines optical flow, Mask R-CNN, and epipolar constraints for dynamic point detection, although it does not consider real-time performance. ACE-Fusion [31] employs an instance segmentation algorithm based on a Dynamic Neural Network (DNN) structure, combined with optical flow, to accurately detect the edges of dynamic objects. However, it does not utilize the surface features of objects when they are stationary for optimizing camera poses. RSO-SLAM [32] proposes an algorithm based on KMC (K-means + Connectivity) that seamlessly integrates semantic information with optical flow information to detect moving regions in the scene, followed by precise motion probability calculation through optical flow decay propagation. VDO-SLAM [33] uses instance segmentation and dense optical flow to accurately identify and track dynamic objects, integrating robot pose, dynamic objects, and static structures into a unified VSLAM framework.

In summary, the existing solutions exhibit several key issues: 

First, they do not account for the presence of moving rigid objects. Dynamic VSLAM approaches that focus on removing dynamic features may result in an insufficient number of remaining static feature points after the dynamic points are removed, which leads to decreased stability and localization accuracy in the VSLAM system.

Second, most methods for testing dynamic feature points based on geometric information rely on constraints such as epipolar lines, fundamental matrix estimation, or reprojection errors. However, each of these constraint methods has its own limitations. For instance, using the distance from matching points to the corresponding epipolar line to determine true dynamicity fails when the moving object travels along the direction of the epipolar line. Additionally, methods that calculate reprojection errors on the imaging plane lose dynamic information in the depth direction.

## 3. Overall System Framework

**Figure 2:**
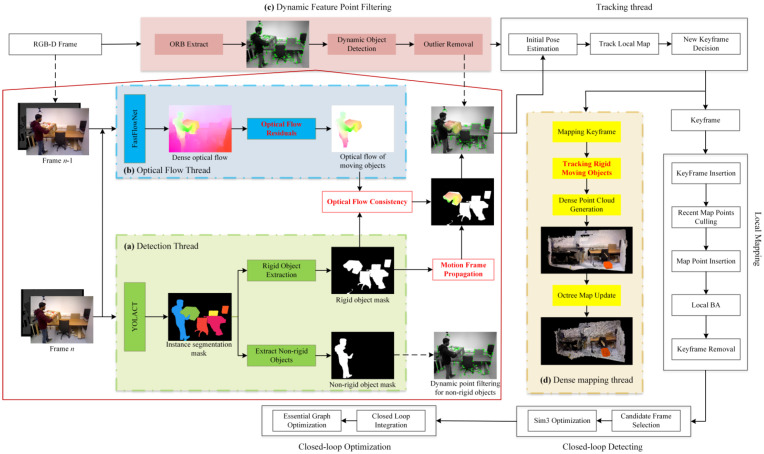
The overall system framework of DIO-SLAM. Key innovations are highlighted in red font, while the original ORB-SLAM3 framework is represented by unfilled boxes. (**a**) Detection thread, represented by green boxes. (**b**) Optical flow thread, represented by blue boxes. (**c**) Dynamic feature point filtering module, which is composed of both the detection and optical flow threads. (**d**) Independent dense mapping thread.

As illustrated in Figure 2, the overall framework of the DIO-SLAM algorithm is presented. The extraction of ORB features follows the same procedure as in the original ORB-SLAM3, extracting both static and dynamic feature points. The algorithm introduces an additional dynamic feature point filtering module atop the original main thread. This filtering module comprises a detection thread and an optical flow thread, which collaborate to effectively remove dynamic feature points. Initially, the detection thread processes the current RGB frame through the YOLACT [34] network for instance segmentation, detecting the masks of all potential moving objects and separately isolating the masks of non-rigid objects. Feature points on non-rigid objects are then removed. Subsequently, the parallel optical flow thread inputs RGB images from the previous and current frames into the FastFlowNet [35] network for real-time optical flow estimation. This method removes the camera’s self-motion flow by calculating optical flow residuals, thereby isolating the optical flow of moving objects. Following this, optical flow consistency is employed to evaluate the motion of all rigid object masks, leading to the identification of masks for moving rigid objects. The feature points on these moving rigid objects are then removed. Finally, a nonlinear optimization method is utilized to track the 6D pose of moving rigid objects, which is then integrated into the dense mapping thread. When the system detects a dynamic frame, the motion frame propagation method can be employed to mitigate errors caused by missed detections. Notably, when a moving rigid object ceases movement, the optical flow thread will respond accordingly, causing the optical flow region of the moving rigid object to disappear, reverting its state to that of a potentially moving rigid object, thus allowing it to re-engage in dense reconstruction and camera pose estimation.

## 4. Methodology Overview

### 4.1. Mask Extraction in the Detection Thread

Compared to object detection algorithms, instance segmentation not only identifies the category of objects but also precisely delineates the pixel-level contours of each object, represented by masks. In dynamic VSLAM, accurate contour information enhances the understanding and differentiation of various objects within the scene, enabling more precise removal of dynamic feature points and thereby improving the localization accuracy of VSLAM algorithms.

YOLACT is a real-time instance segmentation network put forward by Daniel and others from the University of California. Compared to Mask R-CNN, it achieves true real-time segmentation. In this paper, the YOLACT network is selected to segment the instance masks of each input image. Since SLAM systems are often used in unknown environments, the COCO [36] dataset is used for training.

Based on the segmented semantic information, humans, cats, dogs, horses, sheep, cows, elephants, bears, zebras, and giraffes are pre-classified as non-rigid objects, meaning they are considered dynamic in any scenario. Vehicles such as bicycles, cars, motorcycles, airplanes, buses, trains, trucks, and boats, along with common household items like chairs, televisions, computers, keyboards, mice, trash cans, and books, are categorized as rigid objects with the potential to move. Additionally, we included balloons, boxes, rackets, beverage bottles, and school bags in the instance segmentation network training, classifying them as rigid objects.

**Figure 3:**
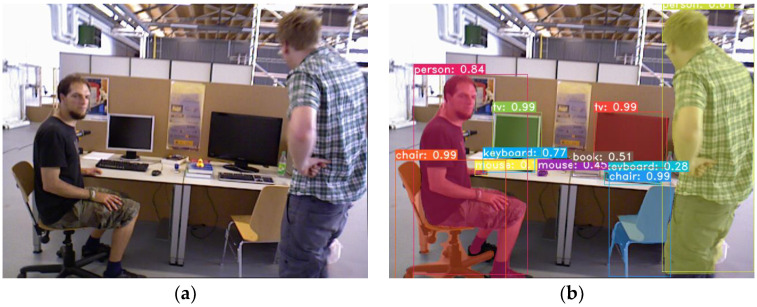
Instance segmentation results and non-rigid object mask extraction. (**a**) RGB frame used for segmentation. (**b**) Instance segmentation output.

It is assumed that the above 30 categories cover most of the dynamic objects in the environment. The instance segmentation thread is designed as a relatively independent module, allowing for the addition of other dynamic objects by retraining the network with new training data. As shown in Figure 3a, a dynamic scene is presented. After performing instance segmentation on the RGB image, object detection boxes, confidence scores, and masks can be obtained, as shown in Figure 3b.

**Figure 4:**
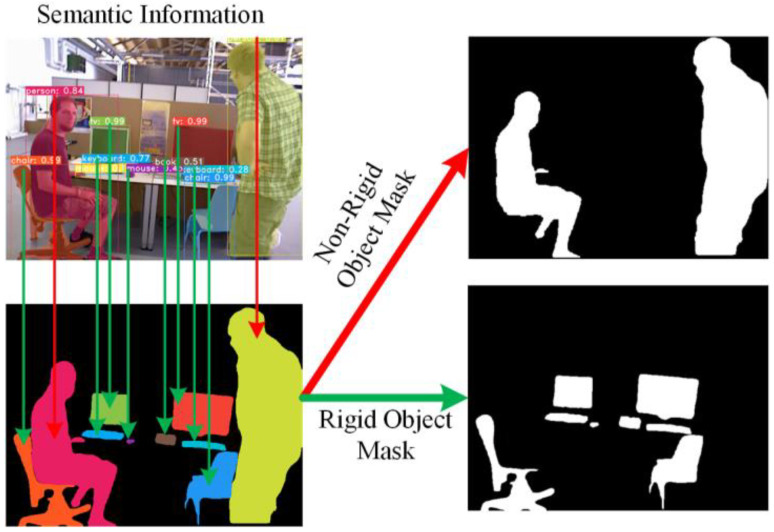
Separation of non-rigid and rigid object masks based on semantic information.

Feature points on non-rigid objects are considered unreliable, while those on rigid objects require further evaluation. Thus, the instance segmentation network segments the content with prior semantic information at the pixel level. Pixels in the rigid object region are assigned a value of 0, and pixels in the non-rigid object region are assigned a value of 1, resulting in the non-rigid object region shown in Figure 4. Conversely, by setting the pixel values of the non-rigid object region to 0 and those of the rigid object region to 1, the rigid object region can be isolated. Let $F_{n}$ represent the RGB image of the n-th frame, and $S_{n}$ represent the instance segmentation result of the n-th frame. The instance segmentation result returned by the instance segmentation thread can be expressed as

(1) $$S_{n}=\{\left(S_{n,id}^{i},S_{n,mask}^{i}\right)|0\leq i\leq \omega \}.$$

In Equation (1), $S_{n,id}^{i}$ and $S_{n,mask}^{i}$ represent the category and mask of the i-th a priori object segmented from $F_{n}$, respectively, where ω denotes the number of instances.

Based on the semantic information, the masks of non-rigid and rigid objects are separated and assigned new labels as follows:

(2) $$S_{n,mask}^{i}\left(x,y\right)=\left\{\begin{array}{cc} k & if\;id\in Non-Rigid\\ k^{\prime } & if\;id\in Rigid \end{array}\right.$$

Equation (2) indicates that when the detected object id belongs to the category of non-rigid objects, the mask is labeled as k. After removing the non-rigid object portion from the mask area, the remaining region corresponds to the rigid object area, labeled as $k^{\prime }$.

### 4.2. Determining Object Motion State in the Optical Flow Thread

In most dynamic scenes, there are not only non-rigid objects like humans and animals that remain in motion for extended periods, but also rigid objects like chairs, books, and boxes that have the potential to move. Typically, these rigid objects remain stationary, with their motion state triggered by human intervention. When in motion, the feature points of such objects need to be removed. Conversely, when stationary, they should be included in feature point extraction to ensure the accuracy of camera pose estimation. For instance, chairs and boxes cannot move on their own but can be moved by people. Clearly, relying solely on semantic information to classify chairs and boxes as static or dynamic objects is insufficient. Semantic static regions do not necessarily correspond to truly static regions; therefore, it is essential to combine semantic information with optical flow to make a more accurate determination.

**Figure 5:**
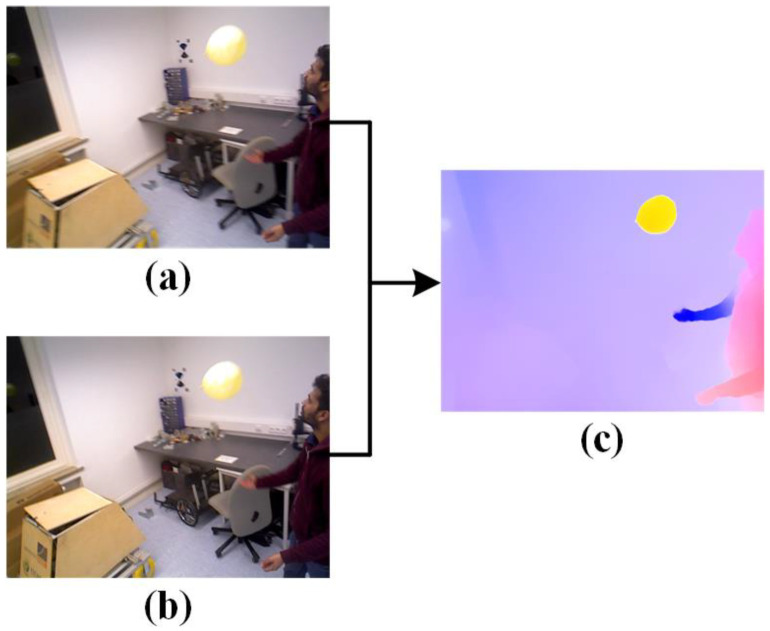
Optical flow network inputs and output. (**a**) Frame n−1. (**b**) Frame n. (**c**) Dense optical flow.

Optical flow can be viewed as the problem of finding motion correspondences for pixels in an image. Given two consecutive RGB frames captured at adjacent times, optical flow estimates the 2D projection field on the image plane [37]. Unlike sparse optical flow, which only targets a limited number of feature points, dense optical flow calculates the displacement of all points in the image, forming a dense optical flow field. This allows for pixel-level image processing using the dense optical flow field. Figure 5 illustrates the optical flow output process for a person repeatedly tossing a balloon. The previous frame n−1 and the current frame *n*, shown in Figure 5a,b, are input into the FastFlowNet network for optical flow estimation, resulting in the dense optical flow map shown in Figure 5c. In the optical flow map, the intensity of the color indicates the motion speed, with lighter pixels representing optical flow caused by the camera’s own movement. In scenarios involving complex camera motion, calculating the camera self-motion flow induced by the camera’s movement is crucial. This type of optical flow reflects the effects of the camera’s movement rather than the independent motion of objects within the scene.

The dense optical flow image comprises both the camera self-motion flow, resulting from camera movement, and the optical flow of moving objects. To eliminate the camera self-motion flow, this paper employs an iterative calculation of optical flow residuals to filter out the noise it generates, thereby isolating the optical flow of moving objects.

**Figure 6:**
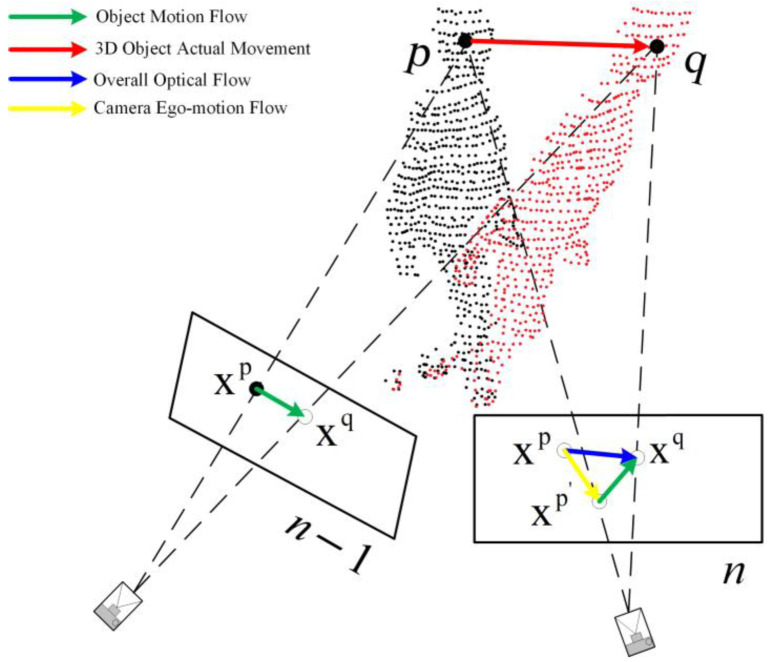
Optical flow changes between adjacent frames.

As shown in Figure 6, the relationship between optical flow changes across different frames is illustrated. The red arrows connecting p and q represent the motion of an object in 3D space, where $x^{p}$ is the pixel coordinate corresponding to the 3D point p of the moving object in frame n−1, and $x^{q}$ is the pixel coordinate corresponding to the 3D point q in frame n. The blue arrow represents the overall optical flow $f_{n-1,n}$, which is derived from the FastFlowNet optical flow calculation network and includes both the camera self-motion flow and the object motion flow. The yellow arrow represents the camera self-motion flow $e_{n-1,n}$. The green arrow is defined as the object motion flow $O_{n-1,n}$. The object motion flow $\vec{x^{p^{\prime }}x^{q}}$ is

(3) $$\vec{x^{p^{\prime }}x^{q}}=\vec{x^{p}x^{q}}-\vec{x^{p}x^{p^{\prime }}}.$$

In Equation (3), through camera pose transformation, the pixels $\;x^{t}\notin k$ outside the non-rigid object in frame n−1 can be projected onto frame n, and the camera self-motion flow $e_{n-1,n}$ is calculated as

(4) $$e_{n-1,n}=x^{t}-\pi \left[T_{n-1}^{-1}\cdot T_{n}\cdot \pi^{-1}\left(x^{t}\right)\right].$$

In Equation (4), π represents the projection of a point from 3D space onto a 2D plane, and $\pi^{-1}$ represents the back-projection of the 2D pixel $x^{t}$ into 3D space. $T_{n-1}^{-1}$ is the inverse of the camera pose matrix at time n−1, and $T_{n}$ is the camera pose matrix at time n. Finally, the object motion flow can be calculated as shown in Equation (5):

(5) $$O_{n-1,n}=\|f_{n-1,n}-e_{n-1,n}{\|}_{2}$$

that is, the optical flow residual $O_{n-1,n}$ is obtained by taking the norm of the difference between the overall optical flow $f_{n-1,n}$ and the camera self-motion flow $e_{n-1,n}$.

**Figure 7:**
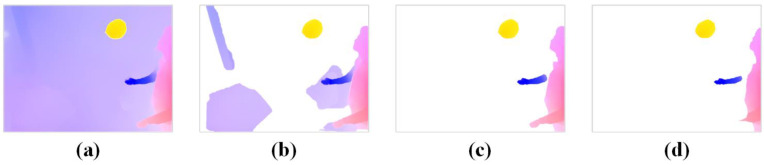
Iterative removal of camera self-motion flow using optical flow residuals. (**a**) Original dense optical flow. (**b**) Number of iterations = 5. (**c**) Number of iterations = 7. (**d**) Number of iterations = 9.

With each iteration, a portion of the camera’s self-motion flow is removed based on the camera’s pose transformation. After multiple iterations, the camera self-motion flow is eliminated, resulting in the motion object optical flow shown in Figure 7. It can be observed that after 5 iterations, some noise remains in the scene. At 7 iterations, the optical flow noise is effectively removed, with the loss rate of the motion object’s optical flow within 10–15%. At 9 iterations, some of the optical flow on the moving object is removed, with a loss rate of about 30%. The calculation method for the optical flow loss rate ${L_{O}}^{p}$ is as follows:

(6) $${L_{O}}^{p}=\frac{{I_{O}}^{p}}{{R_{O}}^{p}}\times 100\%$$

In Equation (6), ${I_{O}}^{p}$ represents the optical flow pixel region after iteration, and ${R_{O}}^{p}$ represents the actual optical flow pixel region of the moving object. It can be concluded that selecting 7 iterations is optimal for extracting the motion object’s optical flow.

### 4.3. Optical Flow Consistency

**Figure 8:**
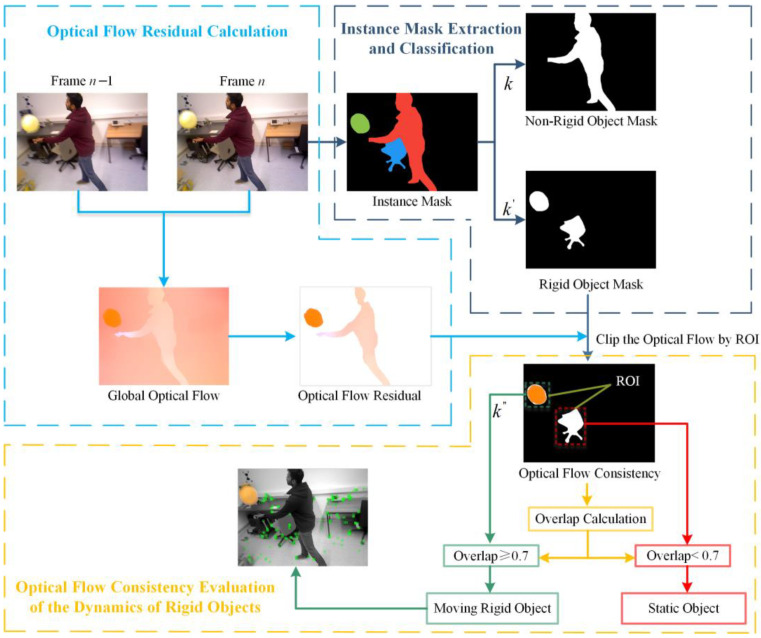
Optical flow consistency for determining the moving rigid object region.

To calculate the true motion state of rigid objects, this paper proposes an optical flow consistency method. The core idea of this method is to use the rigid object masks obtained from instance segmentation to mask specific regions in the optical flow image. Then, image matching methods are used to extract structural features in the optical flow image that are similar to the mask, ensuring these regions are not processed or included in parameter calculations, thereby facilitating the processing or analysis of non-masked regions. The process of using optical flow consistency to determine the motion state of rigid object regions is shown in Figure 8. In the instance segmentation thread, regions labeled as k are excluded, while the mask regions of rigid objects labeled as $k^{\prime }$ are retained. These regions are then combined with the motion object optical flow regions output by the optical flow thread. Let the rigid object mask segmented in frame n be denoted as $S_{n,mask}^{Rigid}$, which is the set of all potential moving rigid object masks. The motion object optical flow region obtained from the optical flow residuals is denoted as $O_{n-1,n}$, where all pixels are dynamic. The rigid object mask region is designated as the region of interest (ROI), and the motion object optical flow region is the image area to be processed, with the pixel set represented as

(7) $$\left\{\begin{array}{c} {\left\{p_{i}\right\}}_{i=1}^{N}\in S_{n,mask}^{Rigid}\\ {\left\{q_{j}\right\}}_{j=1}^{M}\in \;O_{n-1,n}\; \end{array}\right.$$

In Equation (7), N and M represent the number of pixels in the rigid object mask region and the object motion optical flow region, respectively. $p_{i}$ and $q_{j}$ denote the pixels in these two regions.

The region of interest is multiplied by the object motion optical flow region to obtain the optical flow consistency image. The pixel values within the optical flow consistency image region remain unchanged, while the pixel values outside this region are set to 0 (displayed as black). The intersection of the two pixel sets is given by

(8) $${\left\{p_{i}\right\}}_{i=1}^{N}⋂{\left\{q_{j}\right\}}_{j=1}^{M}={\left\{r_{k}\right\}}_{k=1}^{L}$$

In Equation (8), $r_{k}$ represents the intersection of the two pixel sets, where L is the number of intersecting pixels.

The overlap ratio can be expressed as the ratio of the number of intersecting pixels to the pixel count of the smaller region:

(9) $$Overlap=\frac{L}{\min\left(N,M\right)}$$

In Equation (9), if the Overlap ratio is greater than or equal to the threshold of 0.7, the rigid object mask with the highest overlap ratio is considered the moving rigid object mask $S_{n,mask}^{Mv-Rigid}$. If the optical flow consistency check identifies a moving rigid object mask, it is labeled as $k^{\prime \prime }$.

The threshold of 0.7 is suitable for most dynamic scenes. In such scenarios, the overlap ratio between the mask of the moving rigid object and the object motion optical flow region, calculated through optical flow residuals, can indicate whether the object is truly in motion. Extensive experiments and data analysis have shown that when the overlap ratio reaches or exceeds 0.7, the accuracy of motion detection is highest. This effectively separates and removes the feature points of moving rigid objects, preventing interference with camera pose estimation. Additionally, setting the threshold at 0.7 allows for a certain degree of tolerance to minor errors and noise in optical flow calculations. This prevents missed detections of truly moving objects due to an overly high threshold, or false positives that could affect the system’s stability and accuracy with a lower threshold. Consequently, the 0.7 overlap ratio threshold is an optimal value, validated through extensive experimentation, that balances detection accuracy and robustness in dynamic scenes. Algorithm 1 illustrates the process of the optical flow consistency method.
**Algorithm 1** Optical flow consistency calculation$1.\;\mathrm{Initialize}\;S_{n,mask}^{Rigid}\in k^{\prime }$ # Set the rigid object mask region as the ROI region. $2.\;\mathrm{Initialize}\;\;O_{n-1,n}$ #Set the optical flow region as the processing image region. $3.\;\mathrm{Define}\;\mathrm{Function}:\;\mathrm{extract}\_\mathrm{roi}(S_{n,mask}^{Rigid},O_{n-1,n}$)       # Ensure the mask is binary        $S_{n,mask}^{Rigid}=S_{n,mask}^{Rigid}/255$
       # Get the pixels from the instance mask region        ${\left\{p_{i}\right\}}_{i=1}^{N}\leftarrow$*GetInstanceMaskRegionPixels*$(S_{n,mask}^{Rigid}$)       # Get the pixels from the optical flow region        ${\left\{q_{j}\right\}}_{j=1}^{M}\leftarrow$*GetOpticalFlowRegionPixels*$(O_{n-1,n}$)       # Multiply the two sets of pixels element-wise        $\mathrm{multiply}\_\mathrm{images}({\left\{p_{i}\right\}}_{i=1}^{N}$$,\;{\left\{q_{j}\right\}}_{j=1}^{M}$)       ${\left\{r_{k}\right\}}_{k=1}^{L}\leftarrow$*GetOpticalFlowConsistencyImage*    Return extract_roi 4. # Calculate Overlap, where L is the number of intersecting pixels.       $Overlap=L/\min\left(N,M\right)$ 5. If Overlap≥0.7 then       
$S_{n,mask}^{Mv-Rigid}\leftarrow$*GetMovingRigidObjectMask*$(k^{\prime \prime }$)    End If

### 4.4. Motion Frame Propagation

Rotation or blurring caused by object or camera movement can lead to missed detections in instance segmentation. To address this issue, motion frame propagation is applied to keyframes. The strategy involves classifying frames with moving rigid object masks as motion frames, while the remaining frames are considered general frames. The location of dynamic object regions in general frames is determined by transforming the moving rigid object mask from the motion frame using the camera pose. Before propagation, feature point matching is performed. After matching, the pixel coordinates of the static feature points from the previous frame are assigned to the current frame:

(10) $$p_{n}\left(x,y\right)=KT_{n-1\to n}d_{n-1}K^{-1}p_{n-1}\left(x,y\right)$$

In Equation (10), K represents the camera’s intrinsic matrix, $T_{n-1\to n}$ denotes the camera pose transformation matrix from frame n−1 to frame n, $d_{n-1}$ corresponds to the depth value of the pixel, $p_{n-1}\left(x,y\right)$ and $p_{n}\left(x,y\right)$ represent the pixel coordinates in the previous frame and the current frame, respectively.

**Figure 9:**
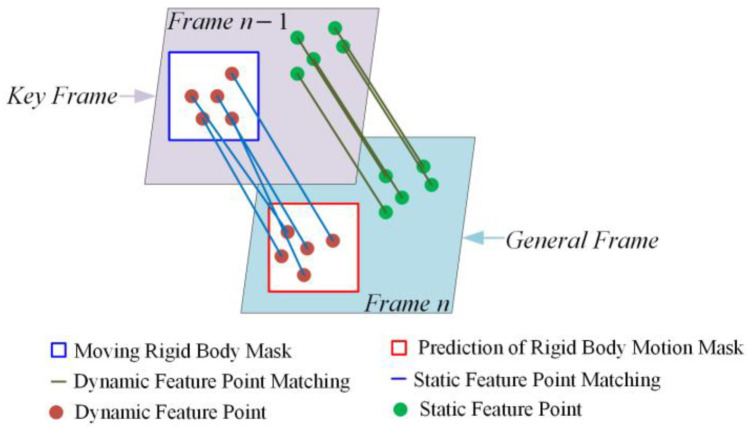
Motion frame propagation.

As shown in Figure 9, the diagram illustrates motion frame propagation. The blue box represents feature points located within the moving rigid object mask, which exhibit dynamic properties. The remaining green points represent feature points on static objects, which exhibit stationary properties. If frame n−1 is designated as a motion frame, the dynamic feature point properties of frame n−1 need to be propagated to the current general frame n. The propagation method is as follows:

(11) $$A\left(p_{n-1}\left(x,y\right)\right)=A\left(p_{n}\left(x,y\right)\right),p_{n-1}\left(x,y\right)↔p_{n}\left(x,y\right)$$

In Equation (11), A represents the attribute, and ↔ indicates the matching relationship. The closer the Euclidean distance between the pixel coordinates of the feature points in consecutive frames and the smaller the difference in depth values, the higher the similarity of the feature points. As a result, the location of the mask in frame n can be inferred using the camera pose transformation matrix. If the moving rigid object mask still exists in frame n, this frame is updated as a motion frame, and the propagation continues.

After completing the motion frame propagation, all subsequent frames will have mask information for the moving objects. Next, the feature points on all moving objects are removed. For each mask $S_{n,mask}^{i}$, the feature points on the masks labeled k and $k^{\prime \prime }$ are removed, while the remaining points are considered static feature points.

**Figure 10:**
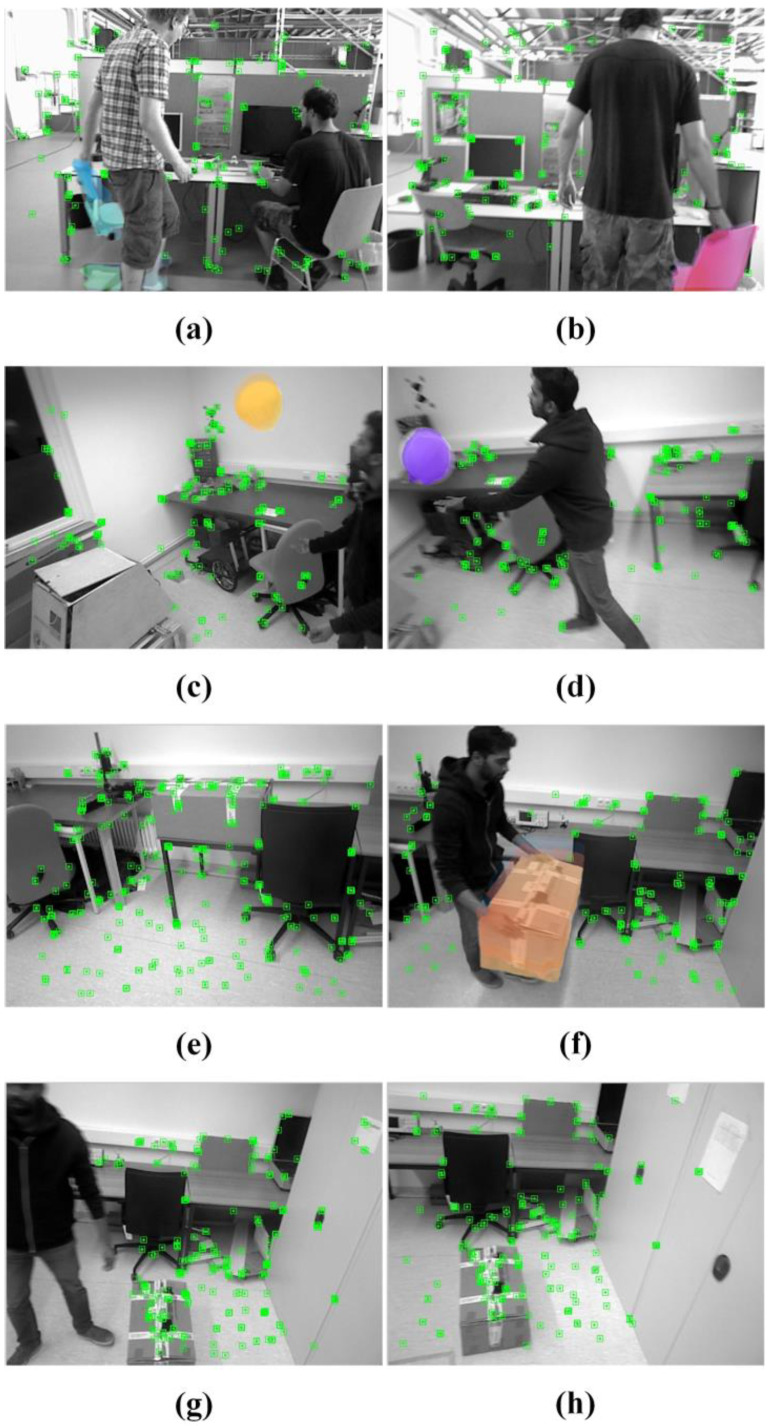
Effect of dynamic feature point removal. The colored areas in the figure depict the optical flow of moving rigid objects, while the green areas indicate the final extracted feature points. The feature points of non-rigid objects, such as the human body, are removed in all scenes. (**a**,**b**) A chair is being dragged, with its feature points being removed. (**c**,**d**) Hitting a balloon, where the feature points on the balloon are removed. (**e**) The box is stationary, and the feature points are normally extracted. (**f**) The box is being moved, with its feature points removed. (**g**,**h**) The box is put down, and its feature points are restored.

The result of removing the dynamic feature points from non-rigid objects and moving rigid objects is shown in Figure 10. The images in Figure 10a–d display dynamic scenes discussed in earlier sections of this paper. It can be observed that the feature points on non-rigid objects, specifically the moving people, have been effectively removed in all four images. Figure 10a,b depict a person dragging a chair and sitting down. Due to the combined effect of the optical flow and detection threads, the rigid objects in the scene are classified as either moving or stationary rigid objects. The chair being dragged is identified as a moving rigid object, and its dynamic feature points are removed. Similarly, Figure 10c,d show the process of a person tossing a balloon and it falling back down. The feature points on the person and the balloon are successfully identified as dynamic points. Notably, in Figure 10c, only part of the person is visible. However, through motion frame propagation, the mask from the previous frame was successfully transformed and transmitted to the next frame using the camera pose transformation. As a result, the dynamic feature points on the incomplete human figure were also effectively removed.

Figure 10e,h illustrate a sequence of images depicting a person moving a stationary box from a table to the floor, with the feature points on the stationary rigid object being retained. In Figure 10e, when the box is stationary, all feature points are extracted normally. In Figure 10f, when a person picks up the box from the table, the detection thread removes the feature points on the person. The optical flow thread then determines that the box is a moving rigid object, leading to the removal of its feature points. In Figure 10g, after the box is placed on the ground and returns to a stationary state, the optical flow thread responds accordingly, and the motion object optical flow disappears. At this point, the detection thread identifies the presence of a non-rigid object, so only the feature points on the person are removed. Finally, as shown in Figure 10h, with no further interference from dynamic objects, all remaining feature points are static and are used for camera pose estimation.

### 4.5. Dense Mapping Thread

Map construction is another key function of VSLAM systems. Compared to dense point clouds, the sparse point clouds generated by current VSLAM solutions are primarily used for robot localization and cannot provide high-level environmental description information. Additionally, they do not account for moving objects in the environment, leading to ghosting effects and reduced map quality. This paper incorporates a dense point cloud reconstruction thread into the original framework, using the keyframes generated by ORB-SLAM3 as the foundation for constructing the point cloud map.

For non-rigid objects, the dynamic regions in the image are segmented based on the original RGB-D camera input, and the static regions are used to generate the point cloud. But simply separating the relevant regions based on semantic information is insufficient for moving rigid objects, as these objects may return to a stationary state after moving. Hence, it is necessary to track their pose and update the position of the moving rigid objects in the point cloud.

Inspired by the work in [38], the motion model is converted into a 6D pose trajectory for each moving rigid object in both the camera and global reference frames. Using traditional visual odometry (VO) for batch estimation to estimate the inliers of each motion model can only yield the trajectory of the camera relative to the moving object, denoted as ego-motion $T_{M_{n}M_{1}}^{ego}$, rather than the trajectory of the moving object in the global reference frame.

Thus, the pose in the first camera frame is used as the global reference frame, with the camera’s motion represented by $T_{c}$. The motion trajectories of the camera and the moving object in the global reference frame are estimated. The estimated poses are then used to project the 3D visual features onto the first frame, allowing for the calculation of the centroid of the surface point set of the moving object. The initial transformation between the moving object and the camera is denoted as $T_{ini}$. The centroid is updated over time as new points are generated through transformations between frames, yielding the motion of each moving object in the global reference frame $T_{M_{n}M_{1}}$:

(12) $$T_{M_{n}M_{1}}=T_{C_{n}C_{1}}T_{M_{n}M_{1}}^{ego}T_{init}^{-1}$$

In Equation (12), $T_{M_{n}M_{1}}^{ego}$ represents the relative motion between the camera and the moving object, and $T_{C_{n}C_{1}}$ denotes the camera’s pose at frame n.

Let the moving rigid object mask region obtained through optical flow consistency in Section 4.3 be denoted as $S_{n,mask}^{Mv-Rigid}$, and the corresponding RGB image frame as $R_{n}^{Mv-Rigid}$. The reconstruction of the point cloud for the moving object is performed by combining $R_{n}^{Mv-Rigid}$ with the depth image $D_{n}$ corresponding to the current frame n. According to the camera imaging principle, the coordinates $\left(x,y,z\right)$ of a point on the moving object in 3D space are given by

(13) $$\left\{\begin{array}{c} x=\left(u-c_{x}\right)/f_{x}\\ y=\left(v-c_{y}\right)/f_{y}\\ z=d \end{array}\right.$$

In Equation (13), $\left(u,v\right)$ represents the pixel coordinates on the $R_{n}^{Mv-Rigid}$ image. The parameters $f_{x}$, $f_{y}$, $c_{x}$, and $c_{y}$ are the camera intrinsics, obtained through camera calibration, and will not be further elaborated here. After obtaining the point cloud of the moving rigid object, the point clouds from consecutive frames are stitched together using the ego-motion transformation $T_{M_{n}M_{n-1}}^{ego}$ between two adjacent frames. When the rigid object is in motion, its point cloud is updated by stitching the new point cloud, and the rigid transformation $T_{M_{n}M_{1}}$ is used to transform the moving rigid object into the global reference frame. For better visual results, the point clouds between frames are aligned using the Iterative Closest Point (ICP) algorithm. After alignment, the point cloud data is processed using an outlier removal filter and a voxel grid downsampling filter. The core of the ICP algorithm involves continuous iteration, where points in two point clouds are rotated and translated within a specified threshold to achieve registration. Since precise registration requires the point clouds to be closely aligned, a coarse registration is performed first to roughly merge the two point clouds, followed by fine registration to further reduce errors. The static background point cloud data is then transformed into the world coordinate system for point cloud stitching and global map fusion, resulting in the generation of a dense point cloud map. Assuming the point clouds generated by the i-th and j-th keyframes are $Cloud_{i}$ and $Cloud_{j}$, and the camera poses are $T_{i}$ and $T_{j}$, the transformation of the keyframes into the world coordinate system is given by Equation (14):

(14) $$\left\{\begin{array}{c} x=\left(u-c_{x}\right)/f_{x}\\ y=\left(v-c_{y}\right)/f_{y}\\ z=d \end{array}\right.$$

Then, the new point cloud is obtained by stitching them together as shown in Equation (15):

(15) $$Cloud^{*}=Clou{d_{i}}^{\prime }+Clou{d_{j}}^{\prime }$$

By stitching the point cloud data generated from the keyframes, a dense point cloud map can be obtained.

## 5. Experiments and Results Analysis

### 5.1. Hardware and Software Platform

The hardware and software configuration of the PC used for testing is shown in Table 1. The training and testing of the instance segmentation and optical flow estimation networks were implemented in Python, with the deep learning framework being PyTorch. To meet real-time requirements, the system converted the deep learning models to ONNX format and used TensorRT and CUDA for accelerated computation. The SLAM components were implemented in C++14, with the point cloud processing using the PCL and OctoMap libraries. The experiments also included several third-party libraries used by the original ORB-SLAM3, such as Eigen3, g2o, Pangolin, DBoW2, and Sophus.

### 5.2. Comparative Experiment on Camera Pose Accuracy with ORB-SLAM3

This paper utilizes the TUM [39] open dataset to verify the accuracy of camera poses, as it includes standard trajectories and comparison tools, making it highly suitable for SLAM-related research. The fr3_walking and fr3_sitting_static subsequences under the fr3 sequence were selected as the experimental datasets. The fr3_walking sequence is highly dynamic, while the fr3_sitting_static sequence has lower dynamics. Sequence fr3_walking also includes four types of camera movements: (1) xyz: the camera moves along the x, y, and z axes; (2) static: the camera position remains fixed; (3) rpy: the camera rotates along the roll, pitch, and yaw axes; (4) halfsphere: the camera moves along a hemisphere with a diameter of 1 m.

The common metrics for evaluating the localization accuracy of SLAM systems are absolute trajectory error (ATE) and relative pose error (RPE). ATE assesses global consistency by measuring the absolute distance between the estimated and true camera poses for each frame. The smaller the value, the higher the accuracy of the camera pose, as it considers only the translation error. RPE calculates the difference in pose change between the estimated and true camera poses over a fixed time interval, taking into account both translation and rotation errors. Root Mean Square Error (RMSE) and Standard Deviation (S.D.) are used as specific quantitative metrics for ATE and RPE. The RMSE value is often influenced by large unexpected errors, while the S.D. value highlights the stability of the SLAM system. The formula for calculating the improvement in accuracy is

(16) $$\eta =\frac{\alpha -\beta }{\alpha }\times 100\%$$

In Equation (16), α represents the results of the algorithm used for comparison, and β represents the results of the algorithm put forward in this paper.

**Figure 11:**
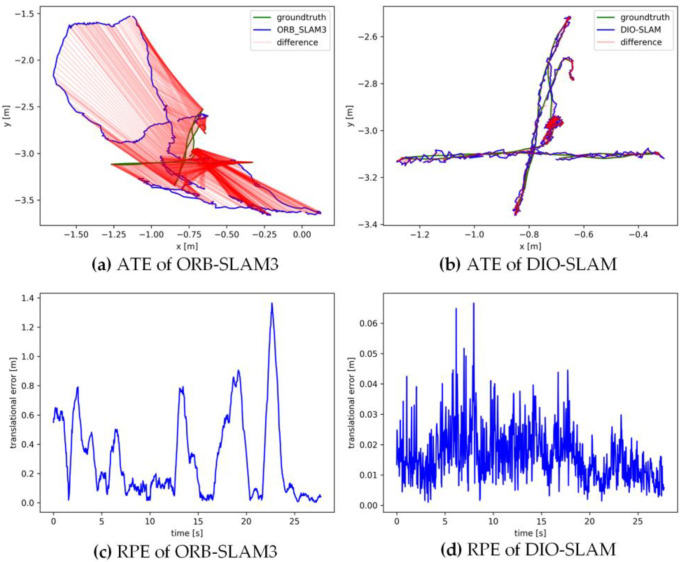
Absolute trajectory error and relative pose error of fr3_walkingx_xyz.

**Figure 12:**
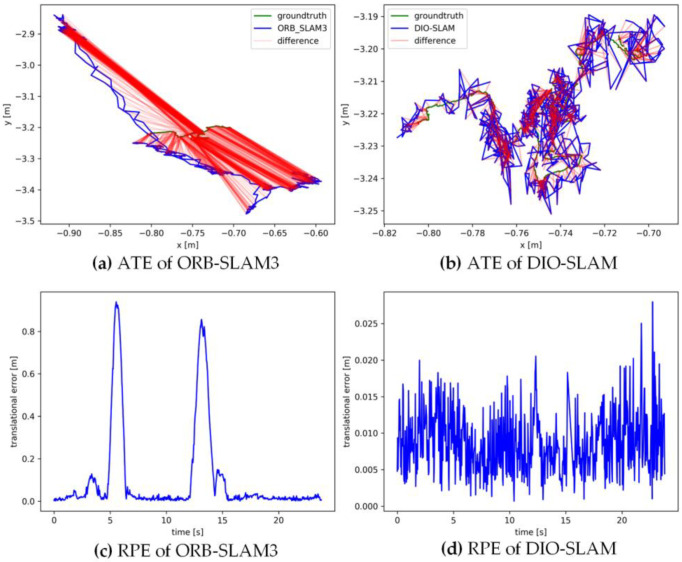
Absolute trajectory error and relative pose error of fr3_walking_static.

**Figure 13:**
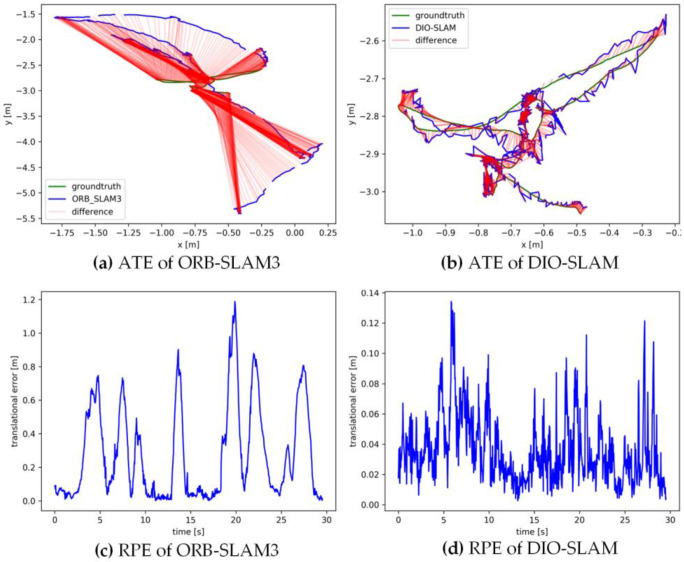
Absolute trajectory error and relative pose error of fr3_walking_rpy.

**Figure 14:**
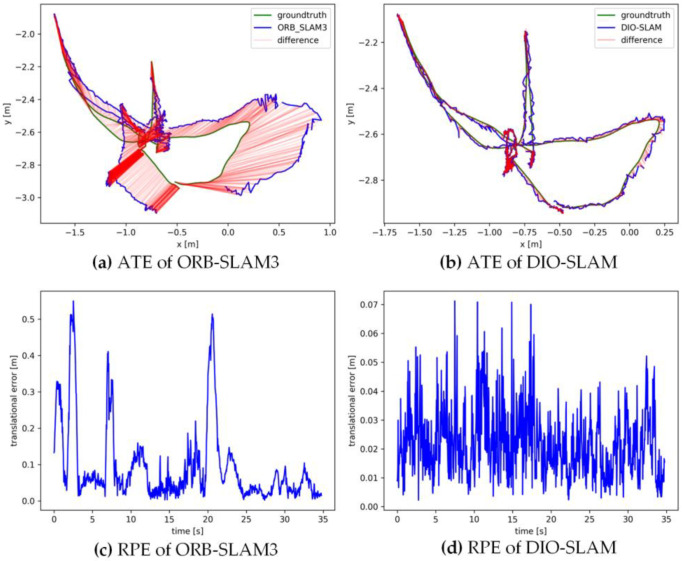
Absolute trajectory error and relative pose error of fr3_walking_halfsphere.

**Figure 15:**
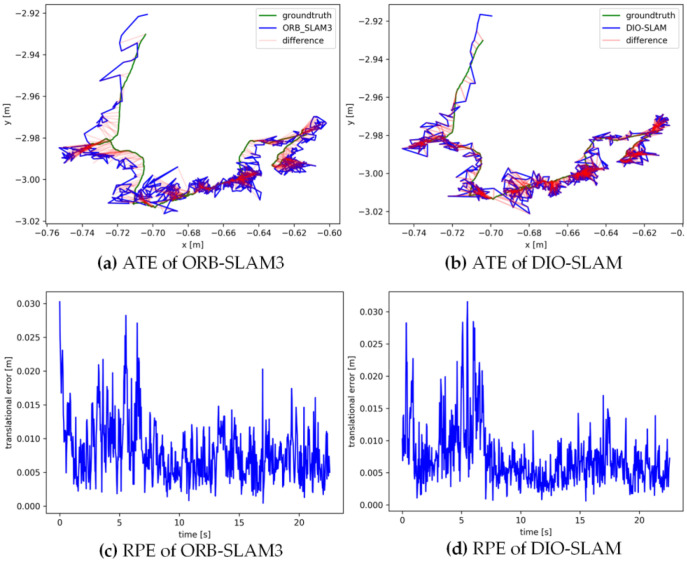
Absolute trajectory error and relative pose error of fr3_sitting_static.

Figure 11, Figure 12, Figure 13, Figure 14 and Figure 15 sequentially display the data comparison between ORB-SLAM3 and DIO-SLAM on five dynamic sequences. In each figure, (a) and (b) show the absolute trajectory error of the algorithm, while (c) and (d) depict the relative pose error. All data were obtained using the same equipment. The green line in the figures represents the ground truth, the blue line represents the estimated trajectory by the algorithm, and the red line indicates the distance between the ground truth and the estimated value, which is also the absolute trajectory error. Shorter red segments indicate smaller errors and higher algorithm accuracy. In the high-dynamic sequences shown in Figure 11, Figure 12, Figure 13 and Figure 14, the camera motion trajectory obtained by our method aligns more closely with the true trajectory, demonstrating that our method can better handle high-dynamic scenes. In all four datasets, there were actions such as pedestrians walking or dragging chairs, which activated the optical flow and instance segmentation threads, successfully eliminating the interference of dynamic points. In the fr3_walking_rpy and fr3_walking_halfsphere sequences, where camera movement was more extensive, the motion frame propagation method compensated for missed detections in the instance segmentation, improving pose accuracy. In contrast, the ORB-SLAM3 algorithm suffered from inaccurate localization due to the presence of dynamic objects, even producing incorrect trajectories in some regions. In the low-dynamic sequence fr3_sitting_static shown in Figure 15, the results of our algorithm are similar to those of the ORB-SLAM3 system, indicating limited room for improvement.

Table 2, Table 3 and Table 4 present the quantitative data comparing the DIO-SLAM and the ORB-SLAM3 algorithm. In the first four high-dynamic sequences, the pose estimation accuracy of the proposed system improved significantly, with the RMSE value of the absolute trajectory error improving by 91.85% to 98.26%. The RMSE value of the translation component of the relative pose error improved by 82.44% to 95.92%, while the rotation component improved by 68.82% to 93.74%. In the fifth low-dynamic sequence, where the movements were limited to slight body twists and hand gestures without moving rigid objects, the low dynamics of the scene did not fully utilize the role of optical flow in the algorithm. With very few dynamic feature points, ORB-SLAM3 was able to handle the situation with its RANSAC algorithm, retaining more static feature points for optimization. Although optical flow did not play a significant role, the instance segmentation in the detection thread was still able to identify the moving person and remove some unstable feature points. Nevertheless, the motion frame propagation process may have led to some stationary objects (such as parts of the human body) being incorrectly classified as moving, reducing the number of feature points participating in pose optimization. As a result, the S.D. value of the relative pose error is slightly lower than that of the ORB-SLAM3 algorithm.

### 5.3. Comparative Experiment on Pose Accuracy with Cutting-Edge Dynamic VSLAM Algorithms

To further validate our algorithm, DIO-SLAM is compared with several leading dynamic scene processing algorithms, including Dyna-SLAM, DS-SLAM, RDMO-SLAM [40], DM-SLAM, RDS-SLAM [41], ACE-Fusion, and SG-SLAM [42]. The results are shown in Table 5, Table 6 and Table 7. 

From the tables, it can be analyzed that the first tier is DIO-SLAM. The introduced optical flow consistency method fully considers objects that are manually moved, and after dynamic frame propagation, it can effectively optimize scenes with significant camera or dynamic object movement. For instance, in the fr3_walking_rpy sequence, most algorithms perform poorly due to the larger camera movements compared to other sequences. Semantic or instance segmentation methods are prone to misjudgment or missed detections of objects in this sequence. DIO-SLAM effectively addresses this issue, achieving the highest accuracy and stability in most dynamic environments. The second tier is SG-SLAM, which performs best in low-dynamic dataset sequences. This is because SG-SLAM avoids over-reliance on deep learning for dynamic feature detection, primarily depending on geometric information. Additionally, by setting a weight threshold, it effectively classifies objects as dynamic and static, thereby improving pose estimation accuracy in low-dynamic scenes. The third tier includes Dyna-SLAM, DS-SLAM, and ACE-Fusion. DS-SLAM performs worse than Dyna-SLAM in high-dynamic sequences due to its use of the lightweight instance segmentation network SegNet, which achieves real-time performance but with reduced accuracy, leading to segmentation errors. ACE-Fusion combines dense optical flow and instance segmentation well, achieving high accuracy in the fr3_walking_static sequence, where the camera is stationary. However, it does not account for camera self-motion flow, resulting in lower accuracy than DIO-SLAM in other high-dynamic sequences.

### 5.4. Ablation Experiment

The DIO-SLAM algorithm we propose is an enhancement of ORB-SLAM3. To evaluate the impact of each component of the algorithm on the system, the instance segmentation part (Y), optical flow part (O), and motion frame propagation part (M) were added sequentially. Then tested on 16 dynamic scene sequences from the Bonn [43] dataset. These diverse scenes include playing with balloons (sequences 1–4), dense pedestrian traffic (sequences 5–7), and moving boxes (sequences 9–16).

As shown in Table 8, DIO-SLAM (Y + O + M) achieved the highest accuracy in 12 of the sequences, while DIO-SLAM (Y + O) achieved the highest accuracy in 4 sequences. In contrast, the traditional ORB-SLAM3 algorithm performed very poorly in high-dynamic scenes. Compared to DIO-SLAM (Y), which only retains instance segmentation, DIO-SLAM (Y + O), which integrates instance segmentation and optical flow, showed a significant improvement in pose accuracy, demonstrating the effectiveness of the optical flow consistency method in filtering moving rigid objects. However, in the crowd and moving_o_box sequences, there is a slight decrease in accuracy after introducing motion frame propagation. This occurs because, in some frames of these sequences, the camera’s field of view is largely occupied by moving objects, leading to errors in the instance segmentation mask extraction. Consequently, the erroneous mask is propagated to subsequent frames by the motion frame propagation.

### 5.5. Dense Mapping Experiment

In the dense mapping thread, RGB-D keyframes without moving rigid and non-rigid objects are used as input for reconstructing the static point cloud background. The point cloud of moving non-rigid objects is updated by tracking their 6D pose and applying rigid transformations. To validate the tracking effectiveness of moving rigid objects, the fr3_walking_xyz sequence from the TUM dataset and the moving_nonobstructing_box sequence from the Bonn dataset were selected for dense point cloud reconstruction.

**Figure 16:**
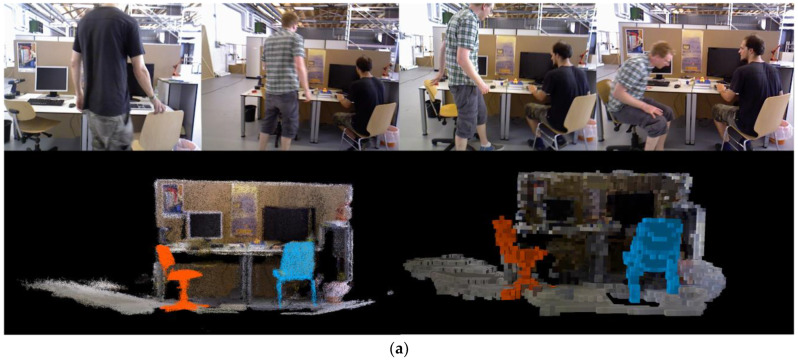

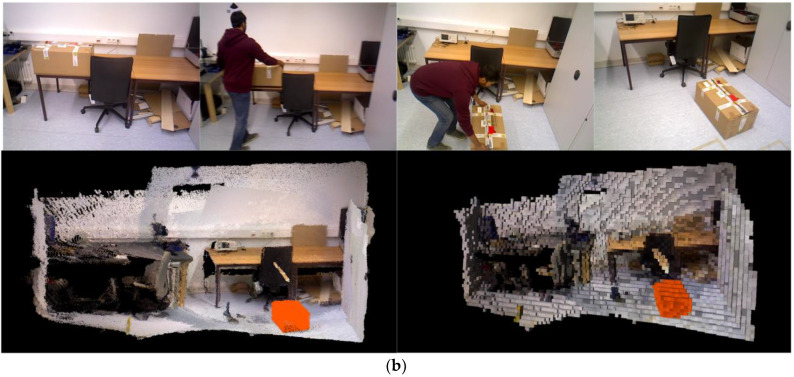
Dense point cloud reconstruction. (**a**) RGB frame, dense point cloud, and octree map of the fr3_walking_xyz sequence. (**b**) RGB frame, dense point cloud, and octree map of the moving_nonobstructing_box sequence.

Figure 16a shows the reconstruction result of the fr3_walking_xyz sequence from the TUM dataset, which includes a moving rigid object—a chair being manually moved. Figure 16b shows the reconstruction result of the moving_nonobstructing_box sequence from the Bonn dataset, where the moving rigid object is a box being carried by a person. Both sequences have removed the non-rigid objects (walking people). The tracked moving object 1 is highlighted with an orange point cloud, and moving object 2 is highlighted with a blue point cloud. The figures demonstrate that the dense mapping thread can accurately reconstruct the point clouds of the static background and stationary rigid objects. The point cloud maps generated for scene 1 and scene 2 are 7.4 MB and 11.5 MB in size, respectively. Such large maps can cause various issues when the algorithm loads or reads data. After storing the maps in octree format, the map sizes are reduced to 98.5 KB and 183.3 KB, saving 98.7% and 98.4% of storage space, respectively. The algorithm can visualize the reconstructed maps in real time, offering practical value for engineering applications.

**Figure 17:**
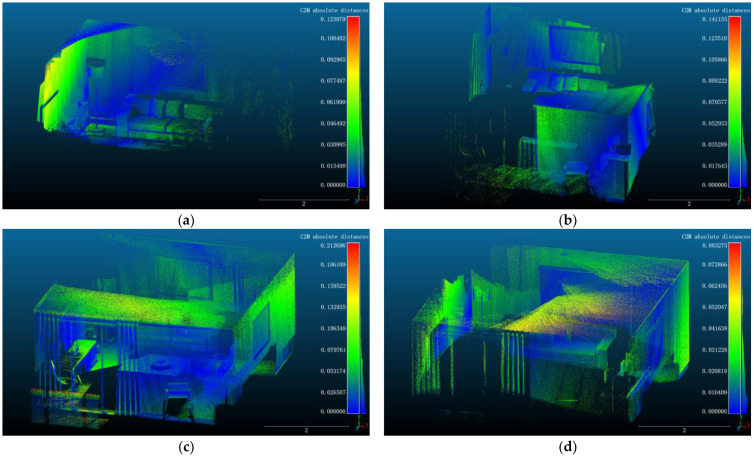
Point cloud error heatmaps. (**a**) kt0 sequence. (**b**) kt1 sequence. (**c**) kt2 sequence. (**d**) kt3 sequence.

To effectively evaluate the quality of the mapping, dense point cloud reconstruction was performed on four datasets from the ICL-NUIM [44] (kt0, kt1, kt2, and kt3). The point clouds reconstructed using DIO-SLAM were aligned with the ground truth 3D models. Figure 17 shows the heatmaps generated after aligning the point cloud maps with the ground truth 3D models. Blue and green indicate high overlap of points, while yellow and red indicate lower overlap.

Additionally, Table 9 presents the mean distance and standard error between the dense point clouds reconstructed by our method and the standard model. The mean distance is the average of all point-to-point distances between the two point clouds, used to measure the overall difference between them. The standard error indicates the distribution of distances; a larger standard error suggests a more uneven distribution of point-to-point distances, while a smaller standard error indicates a more concentrated distribution. The average mean distance across the four sequences is 0.0257 m, and the average standard error is 0.0198 m. This leads to the conclusion that the dense point cloud maps constructed by DIO-SLAM have a high degree of overlap with the ground truth models, accurately reconstructing the real-world scenes.

### 5.6. Real-World Scenario Testing

To validate the effectiveness of the proposed approach in real-world scenarios, this section conducts tests on indoor dynamic scenes using an Orbbec Astra depth camera. The experimental data were processed by recording the camera’s/camera/color/image_raw and/camera/depth/image_raw topics using the rosbag command in the ROS system. The bag files were then converted into the TUM dataset format based on their timestamps.

**Figure 18:**
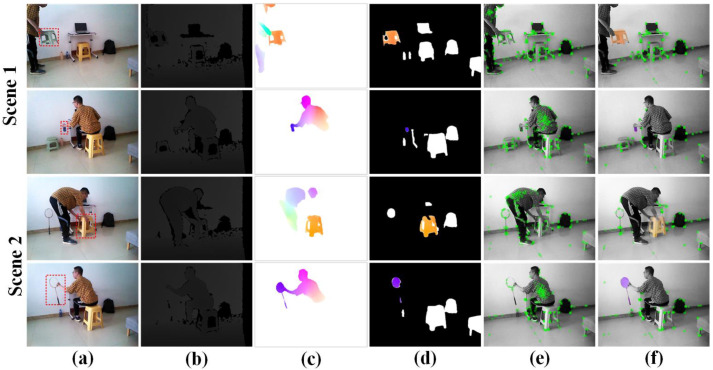
Real-world scenario test results. (**a**) Color images. (**b**) Depth images. (**c**) Optical flow of moving objects. (**d**) Moving rigid object masks. (**e**) Feature points in traditional ORB-SLAM3. (**f**) Feature points in DIO-SLAM.

Figure 18 shows the test results of two real-world scenarios, with the chair in Scenario 1 being replaced by a racket in Scenario 2. Figure 18a presents five color image frames from the dynamic scenes, with dynamic objects outlined in red dashed boxes. In the first row, a person places a chair with large movements and a long duration. In the second row, a person picks up a bottle to drink water, with smaller movements, shorter duration, and a small target object. In the third row, a person moves a stool with large movements and a short duration. In the fourth row, a person picks up a racket with medium movements and medium duration. The corresponding depth images are shown in Figure 18b. The optical flow of moving objects obtained through optical flow residuals is shown in Figure 18c. By combining the optical flow of moving objects with the masks of rigid objects using optical flow consistency, the masks of moving rigid objects, as shown in Figure 18d, are obtained. Figure 18e shows the feature points from ORB-SLAM3, and Figure 18f shows the effect of DIO-SLAM in filtering out dynamic points. It can be seen that DIO-SLAM successfully removes the dynamic feature points associated with the moving human body, the chair, and the racket.

### 5.7. Time Analysis

In terms of running speed, experiments show that YOLACT’s processing time per frame is three times faster than Mask R-CNN, and FastFlowNet reduces the computational cost by 13.4 times (only 12.2 GFLOPS) while achieving 90% of LiteFlowNet’s [45] performance, with the computation time reduced by three times. For this reason, compared to other deep learning-based SLAM algorithms, the parallel architecture of YOLACT and FastFlowNet significantly shortens the processing time of the dynamic detection thread, meeting real-time requirements (approximately 23 frames per second).

Table 10 provides a comparison of computation times between the DIO-SLAM algorithm and other algorithms. Since our method adds a detection thread and an optical flow thread for handling dynamic scenes, its runtime is longer than that of ORB-SLAM3. Nonetheless, compared to Dyna-SLAM, DS-SLAM, and RDS-SLAM, the runtime of DIO-SLAM is significantly reduced. The runtime of DIO-SLAM is similar to that of the latest algorithm, SG-SLAM, but DIO-SLAM offers higher localization accuracy and better applicability in scenarios involving moving rigid objects. Table 11 lists the real-time runtime of each module in DIO-SLAM. After feature point extraction, the FastFlowNet and YOLACT networks are accelerated using CUDA (Compute Unified Device Architecture) and TensorRT, and since they run in parallel, they do not add additional time. Although the optical flow consistency (OFC) module has its own processing time, it is not separately listed or included in the overall per-frame processing time. This is because the OFC module is triggered under specific detection conditions and is not executed for every frame. Therefore, it does not directly affect the overall computation time per frame, especially in static or low-dynamic scenes where the module may not perform any operations, and thus its time is not separately accounted for in the per-frame processing time. Overall, the processing time per frame is approximately 43.10 ms, with about 23 frames per second (FPS), meeting the real-time camera pose estimation and localization requirements for most scenarios. Additionally, the dense point cloud mapping module is an independent optional module in this paper. When this module is enabled, the frame rate is approximately 15–20 frames per second.

## 6. Conclusions

This paper introduces the DIO-SLAM algorithm, designed to achieve a balance between real-time performance and accuracy. Building upon the traditional ORB-SLAM3, the system incorporates a dynamic feature point removal module, which consists of detection and optical flow threads. The detection thread segments masks of potentially moving objects and removes feature points of prior non-rigid objects based on semantic information. Meanwhile, the optical flow thread leverages optical flow residuals to filter out noise caused by the camera’s self-motion flow. The proposed optical flow consistency method integrates the detection and optical flow threads, utilizing the YOLACT algorithm to detect and eliminate non-rigid object feature points. For moving rigid objects, the algorithm uses the optical flow of these objects to identify their masks and remove dynamic feature points. When a moving rigid object becomes stationary, the optical flow thread adjusts accordingly, incorporating its feature points into the camera pose estimation, which significantly enhances accuracy. To address missed detections resulting from large movements of the camera or objects, a motion frame propagation method is introduced, which transfers the motion attributes of frames and feature points to subsequent frames, improving the continuity of object motion across frames. Additionally, the algorithm includes an optional dense mapping thread that tracks the 6D pose of moving rigid objects, updates their positions in the point cloud in real-time through rigid transformations, and stores the point cloud in an octree format. Experiments show that DIO-SLAM excels in highly dynamic scenes and effectively manages significant camera movements. Ablation studies and pose accuracy experiments further confirm its superior localization accuracy and robustness.

The DIO-SLAM algorithm can still be improved in future work. For example, instance segmentation and dense optical flow estimation play crucial roles in the system; using more accurate algorithms while ensuring real-time performance could significantly enhance system performance. Additionally, the algorithm may fail if a moving object occupies a large portion of the camera’s field of view during actual testing. Furthermore, the current algorithm does not incorporate optical flow for determining non-rigid objects. It would be an interesting research direction to explore how to assess the stationary parts of non-rigid objects, such as humans. These challenges are expected to be further studied in future work to develop a more comprehensive and stable algorithmic system.

## Figures and Tables

**Table 1:** Hardware and software configurations of the experimental platform.

Name	Configuration
CPU	Intel Core i9-13900HX (Intel, Santa Clara, CA, USA)
GPU	NVIDIA GeForce RTX 4060 8G (NVIDIA, Santa Clara, CA, USA)
Memory	32GB
Operating system	Ubuntu 20.04 64-bit
Python, CUDA, PyTorch versions	python3.8, CUDA = 11.8, pytorch = 2.0.0
OpenCV, PCL versions	OPENCV = 4.8.0, PCL = 1.10
TensorRT version	TensorRT = 8.6.1.6

**Table 2:** Results of metric absolute trajectory error (ATE) [m].

Seq.	ORB-SLAM3		DIO-SLAM (Ours)		Improvements	
	RMSE	S.D.	RMSE	RMSE	RMSE (%)	S.D. (%)
w_xyz	0.8336	0.4761	0.0145	0.0145	98.26	98.53
w_static	0.4078	0.1831	0.0072	0.0072	98.23	98.31
w_rpy	1.1665	0.6294	0.0306	0.0306	97.38	97.47
w_half	0.3178	0.1557	0.0259	0.0259	91.85	91.91
s_static	0.0099	0.0044	0.0062	0.0062	37.37	34.09

**Table 3:** Results of metric translational drift (RPE) [m/s].

Seq.	ORB-SLAM3		DIO-SLAM (Ours)		Improvements	
	RMSE	S.D.	RMSE	RMSE	RMSE (%)	S.D. (%)
w_xyz	0.4154	0.2841	0.0186	0.0089	95.52	96.87
w_static	0.2355	0.2123	0.0096	0.0041	95.92	98.07
w_rpy	0.3974	0.2836	0.0426	0.0228	89.28	91.96
w_half	0.1424	0.1075	0.0250	0.0110	82.44	89.77
s_static	0.0092	0.0045	0.0087	0.0047	5.43	−4.44

**Table 4:** Results of metric rotational drift (RPE) [deg/s].

Seq.	ORB-SLAM3		DIO-SLAM (Ours)		Improvements	
	RMSE	S.D.	RMSE	RMSE	RMSE (%)	S.D. (%)
w_xyz	8.0130	5.5450	0.5020	0.2455	93.74	95.57
w_static	4.1851	3.7462	0.2708	0.1106	93.53	97.05
w_rpy	7.7448	5.5218	0.8731	0.4221	88.73	92.36
w_half	2.3482	1.6014	0.7321	0.3435	68.82	78.55
s_static	0.2872	0.1265	0.2802	0.1287	2.44	−1.74

**Table 5:** ATE comparison of the latest algorithms on 5 sequences.

	Seq.	w_xyz	w_static	w_rpy	w_half	s_static
Dyna-SLAM * (N + G)	RMSE	0.0161	**0.0067**	0.0345	0.0293	0.0108
	S.D.	0.0083	0.0038	0.0195	0.0149	0.0056
DS-SLAM *	RMSE	0.0247	0.0081	0.4442	0.0303	0.0065
	S.D.	0.0161	0.0067	0.2350	0.0159	0.0033
RDMO-SLAM	RMSE	0.0226	0.0126	0.1283	0.0304	0.0066
	S.D.	0.0137	0.0071	0.1047	0.0141	0.0033
DM-SLAM	RMSE	0.0148	0.0079	0.0328	0.0274	0.0063
	S.D.	0.0072	0.0040	0.0194	0.0137	0.0032
RDS-SLAM *	RMSE	0.0571	0.0206	0.1604	0.0807	0.0084
	S.D.	0.0229	0.0120	0.0873	0.0454	0.0043
ACE-Fusion	RMSE	0.0146	**0.0067**	0.1869	0.0425	0.0066
	S.D.	0.0074	0.0032	0.1467	0.0264	0.0032
SG-SLAM *	RMSE	0.0152	0.0073	0.0324	0.0268	**0.0060**
	S.D.	0.0075	0.0034	0.0187	0.0203	0.0047
DIO-SLAM(Ours)	RMSE	**0.0145**	0.0072	**0.0306**	**0.0259**	0.0062
	S.D.	**0.0070**	**0.0031**	**0.0159**	**0.0126**	**0.0029**

* Indicates that the algorithm has open-source code available. The highest accuracy among all algorithms is indicated in bold.

**Table 6:** RPE (translational drift) comparison of the latest algorithms on 5 sequences.

	Seq.	w_xyz	w_static	w_rpy	w_half	s_static
Dyna-SLAM * (N + G)	RMSE	0.0217	**0.0089**	0.0448	0.0284	0.0126
	S.D.	0.0119	0.0040	0.0262	0.0149	0.0067
DS-SLAM *	RMSE	0.0333	0.0102	0.1503	0.0297	0.0078
	S.D.	0.0229	**0.0038**	0.1168	0.0152	0.0038
RDMO-SLAM	RMSE	0.0299	0.0160	0.1396	0.0294	0.0090
	S.D.	0.0188	0.0090	0.1176	0.0130	0.0040
DM-SLAM	RMSE	-	-	-	-	-
	S.D.	-	-	-	-	-
RDS-SLAM *	RMSE	0.0426	0.0221	0.1320	0.0482	0.0123
	S.D.	0.0317	0.0149	0.1067	0.0036	0.0070
ACE-Fusion	RMSE	-	-	-	-	-
	S.D.	-	-	-	-	-
SG-SLAM *	RMSE	0.0194	0.0100	0.0450	0.0279	**0.0075**
	S.D.	0.0100	0.0051	0.0262	0.0146	**0.0035**
DIO-SLAM(Ours)	RMSE	**0.0186**	0.0096	**0.0426**	**0.0250**	0.0087
	S.D.	**0.0089**	0.0041	**0.0228**	0.0110	0.0047

- Indicates missing data and no open-source code. * Indicates that the algorithm has open-source code available. The highest accuracy among all algorithms is indicated in bold.

**Table 7:** RPE (rotational drift) comparison of the latest algorithms on 5 sequences.

	Seq.	w_xyz	w_static	w_rpy	w_half	s_static
Dyna-SLAM * (N + G)	RMSE	0.6284	**0.2612**	0.9894	0.7842	0.3416
	S.D.	0.3848	0.1259	0.5701	0.4012	0.1642
DS-SLAM *	RMSE	0.8266	0.2690	3.0042	0.8142	0.2735
	S.D.	0.2826	0.1215	2.3065	0.4101	0.1215
RDMO-SLAM	RMSE	0.7990	0.3385	2.5472	0.7915	0.2910
	S.D.	0.5502	0.1612	2.0607	0.3782	0.1330
DM-SLAM	RMSE	-	-	-	-	-
	S.D.	-	-	-	-	-
RDS-SLAM *	RMSE	0.9222	0.4944	13.1693	1.8828	0.3338
	S.D.	0.6509	0.3112	12.0103	1.5250	0.1706
ACE-Fusion	RMSE	-	-	-	-	-
	S.D.	-	-	-	-	-
SG-SLAM*	RMSE	0.5040	0.2679	0.9565	0.8119	**0.2657**
	S.D.	0.2469	0.1144	0.5487	0.3878	**0.1163**
DIO-SLAM(Ours)	RMSE	**0.5020**	0.2708	**0.8731**	**0.7321**	0.2802
	S.D.	**0.2455**	**0.1106**	**0.4221**	**0.3435**	0.1287

- Indicates missing data and no open-source code. * Indicates that the algorithm has open-source code available. The highest accuracy among all algorithms is indicated in bold.

**Table 8:** Comparison of ATE on the Bonn dataset.

Seq.		ORB-SLAM3	DIO-SLAM (Y)	DIO-SLAM (Y + O)		DIO-SLAM (Y + O+M)	
		RMSE	RMSE	RMSE	Im(%)	RMSE	Im(%)
1	balloon	0.1762	0.0309	0.0307	0.6	**0.0291**	5.2
2	balloon2	0.2898	0.0312	0.0296	5.1	**0.0284**	4.1
3	balloon_tracking	0.0284	0.0280	0.0272	2.9	**0.0259**	4.8
4	balloon_tracking2	0.1400	0.0992	0.0589	40.6	**0.0568**	3.6
5	crowd	0.6262	0.0289	**0.0289**	-	0.0292	−1.0
6	crowd2	1.5959	0.0297	**0.0297**	-	0.0303	−2.0
7	crowd3	0.9958	0.0281	0.0280	0.3	**0.0274**	2.1
8	moving_no_box	0.2634	0.0294	0.0196	33.3	**0.0173**	11.7
9	moving_no_box2	0.0379	0.0477	0.0314	34.2	**0.0286**	8.9
10	placing_no_box	0.7875	0.0486	0.0201	58.6	**0.0189**	6.0
11	placing_no_box2	0.0283	0.0290	0.0182	37.2	**0.0167**	8.2
12	placing_no_box3	0.2076	0.0656	0.0433	34.0	**0.0388**	10.3
13	removing_no_box	0.0167	0.0161	0.0155	3.7	**0.0140**	9.7
14	removing_no_box2	0.0225	0.0227	0.0224	1.3	**0.0211**	5.8
15	moving_o_box	0.6476	0.2628	**0.2628**	-	0.2633	−0.2
16	moving_o_box2	0.7903	0.1344	**0.1241**	7.7	0.1248	−0.6

- Indicates missing data and no open-source code. The highest accuracy among all algorithms is indicated in bold.

**Table 9:** Mean distance and standard error between the dense point clouds reconstructed by the proposed method and the standard model [m].

Seq.	Mean Distance	Std Deviation
kt0	0.0253	0.0193
kt1	0.0203	0.0142
kt2	0.0378	0.0296
kt3	0.0193	0.0159
Average	0.0257	0.0198

**Table 10:** Time evaluation.

Algorithm	Average Processing Time per Frame (ms)	Hardware Platform
ORB-SLAM3	25.46	Intel Core i9-13900HX Without GPU
Dyna-SLAM ^#^	192.00 (at least)	Nvidia Tesla M40 GPU
DS-SLAM ^#^	59.40	Intel i7 CPU, P4000 GPU
RDS-SLAM ^#^	57.50	Nvidia RTX 2080Ti GPU
SG-SLAM ^#^	65.71	Nvidia Jetson AGX Xavier Developer Kit
SG-SLAM ^#^	39.51	AMD Ryzen 7 4800H (AMD, Santa Clara, CA, USA), Nvidia GTX 1650
DIO-SLAM (Before TensorRT acceleration)	124.80	Intel Core i9-13900HX NVIDIA GeForce RTX 4060 8G
DIO-SLAM (TensorRT acceleration)	43.10	Intel Core i9-13900HX NVIDIA GeForce RTX 4060 8G

# Indicates that the data comes from the original literature.

**Table 11:** Average running time of each module [ms].

Method	ORB Extraction	FastFlowNet (TensorRT Acceleration)	YOLACT (TensorRT Acceleration)	OFC	MFP	Each Frame
Time Cost	3.89	18.39	25.20	5.01	14.01	43.10

## Data Availability

The data that support the findings of this study are available from the corresponding author upon reasonable request. “COCO dataset” at https://cocodataset.org (accessed on 19 August 2024). “TUM dataset” at https://vision.in.tum.de/data/datasets/rgbd-dataset/ (accessed on 19 August 2024). “Bonn dataset” at https://www.ipb.uni-bonn.de/data/rgbd-dynamic-dataset/index.html (accessed on 19 August 2024). “ICL-NUIM dataset” at https://www.doc.ic.ac.uk/~ahanda/VaFRIC/iclnuim.html (accessed on 19 August 2024).

## References

[1] Zheng Z., Lin S., Yang C. (2024). RLD-SLAM: A Robust Lightweight VI-SLAM for Dynamic Environments Leveraging Semantics and Motion Information. IEEE Trans. Ind. Electron..

[2] Jia G., Li X., Zhang D., Xu W., Lv H., Shi Y., Cai M. (2022). Visual-SLAM Classical Framework and Key Techniques: A Review. Sensors.

[3] Chen W., Shang G., Ji A., Zhou C., Wang X., Xu C., Li Z., Hu K. (2022). An Overview on Visual SLAM: From Tradition to Semantic. Remote Sens..

[4] Macario Barros A., Michel M., Moline Y., Corre G., Carrel F. (2022). A Comprehensive Survey of Visual SLAM Algorithms. Robotics.

[5] Tourani A., Bavle H., Sanchez-Lopez J.L., Voos H. (2022). Visual SLAM: What Are the Current Trends and What to Expect?. Sensors.

[6] Zhang F., Rui T., Yang C., Shi J. (2019). LAP-SLAM: A Line-Assisted Point-Based Monocular VSLAM. Electronics.

[7] Mur-Artal R., Tardós J.D. (2017). ORB-SLAM2: An Open-Source SLAM System for Monocular, Stereo, and RGB-D Cameras. IEEE Trans. Robot..

[8] Mur-Artal R., Montiel J.M.M., Tardós J.D. (2015). ORB-SLAM: A Versatile and Accurate Monocular SLAM System. IEEE Trans. Robot..

[9] Campos C., Elvira R., Rodríguez J.J.G., Montiel J.M.M., Tardós J.D. (2021). ORB-SLAM3: An Accurate Open-Source Library for Visual, Visual–Inertial, and Multimap SLAM. IEEE Trans. Robot..

[10] Aad G., Anduaga X.S., Antonelli S., Bendel M., Breiler B., Castrovillari F., Civera J.V., Del Prete T., Dova M.T., Duffin S. (2008). The ATLAS Experiment at the CERN Large Hadron Collider. J. Instrum..

[11] Zhong F., Wang S., Zhang Z., Chen C., Wang Y. Detect-SLAM: Making Object Detection and SLAM Mutually Beneficial. Proceedings of the 2018 IEEE Winter Conference on Applications of Computer Vision (WACV).

[12] Liu W., Anguelov D., Erhan D., Szegedy C., Reed S., Fu C.-Y., Berg A.C., Leibe B., Matas J., Sebe N., Welling M. (2016). SSD: Single Shot MultiBox Detector. Proceedings of the Computer Vision—ECCV 2016.

[13] Runz M., Buffier M., Agapito L. MaskFusion: Real-Time Recognition, Tracking and Reconstruction of Multiple Moving Objects. Proceedings of the 2018 IEEE International Symposium on Mixed and Augmented Reality (ISMAR).

[14] He K., Gkioxari G., Dollar P., Girshick R. Mask R-CNN. Proceedings of the 2017 IEEE International Conference on Computer Vision (ICCV).

[15] Whelan T., Leutenegger S., Salas Moreno R., Glocker B., Davison A. ElasticFusion: Dense SLAM Without A Pose Graph. Proceedings of the Robotics: Science and Systems XI.

[16] Yu C., Liu Z., Liu X.-J., Xie F., Yang Y., Wei Q., Fei Q. DS-SLAM: A Semantic Visual SLAM towards Dynamic Environments. Proceedings of the 2018 IEEE/RSJ International Conference on Intelligent Robots and Systems (IROS).

[17] Badrinarayanan V., Kendall A., Cipolla R. (2017). SegNet: A Deep Convolutional Encoder-Decoder Architecture for Image Segmentation. IEEE Trans. Pattern Anal. Mach. Intell..

[18] Sun L., Wei J., Su S., Wu P. (2022). SOLO-SLAM: A Parallel Semantic SLAM Algorithm for Dynamic Scenes. Sensors.

[19] Wang X., Zhang R., Kong T., Li L., Shen C. (2020). SOLOv2: Dynamic and Fast Instance Segmentation. Proceedings of the Advances in Neural Information Processing Systems.

[20] Bescos B., Fácil J.M., Civera J., Neira J. (2018). DynaSLAM: Tracking, Mapping, and Inpainting in Dynamic Scenes. IEEE Robot. Autom. Lett..

[21] Bescos B., Campos C., Tardós J.D., Neira J. (2021). DynaSLAM II: Tightly-Coupled Multi-Object Tracking and SLAM. IEEE Robot. Autom. Lett..

[22] Wang X., Zheng S., Lin X., Zhu F. (2023). Improving RGB-D SLAM Accuracy in Dynamic Environments Based on Semantic and Geometric Constraints. Measurement.

[23] Islam Q.U., Ibrahim H., Chin P.K., Lim K., Abdullah M.Z. (2024). MVS-SLAM: Enhanced Multiview Geometry for Improved Semantic RGBD SLAM in Dynamic Environment. J. Field Robot..

[24] Zhang T., Zhang H., Li Y., Nakamura Y., Zhang L. FlowFusion: Dynamic Dense RGB-D SLAM Based on Optical Flow. Proceedings of the 2020 IEEE International Conference on Robotics and Automation (ICRA).

[25] Sun D., Yang X., Liu M.-Y., Kautz J. PWC-Net: CNNs for Optical Flow Using Pyramid, Warping, and Cost Volume. Proceedings of the IEEE Conference on Computer Vision and Pattern Recognition (CVPR).

[26] Chang Z., Wu H., Sun Y., Li C. (2022). RGB-D Visual SLAM Based on Yolov4-Tiny in Indoor Dynamic Environment. Micromachines.

[27] Zhang X., Zhang R., Wang X. (2022). Visual SLAM Mapping Based on YOLOv5 in Dynamic Scenes. Appl. Sci..

[28] Theodorou C., Velisavljevic V., Dyo V. (2022). Visual SLAM for Dynamic Environments Based on Object Detection and Optical Flow for Dynamic Object Removal. Sensors.

[29] Lucas B.D., Kanade T. An Iterative Image Registration Technique with an Application to Stereo Vision. Proceedings of the IJCAI’81: 7th international joint conference on Artificial intelligence.

[30] Cheng J., Wang Z., Zhou H., Li L., Yao J. (2020). DM-SLAM: A Feature-Based SLAM System for Rigid Dynamic Scenes. ISPRS Int. J. Geo-Inf..

[31] Bujanca M., Lennox B., Luján M. ACEFusion—Accelerated and Energy-Efficient Semantic 3D Reconstruction of Dynamic Scenes. Proceedings of the 2022 IEEE/RSJ International Conference on Intelligent Robots and Systems (IROS).

[32] Qin L., Wu C., Chen Z., Kong X., Lv Z., Zhao Z. (2024). RSO-SLAM: A Robust Semantic Visual SLAM With Optical Flow in Complex Dynamic Environments. IEEE Trans. Intell. Transp. Syst..

[33] Zhang J., Henein M., Mahony R., Ila V. (2021). VDO-SLAM: A Visual Dynamic Object-Aware SLAM System. arXiv.

[34] Bolya D., Zhou C., Xiao F., Lee Y.J. YOLACT: Real-Time Instance Segmentation. Proceedings of the IEEE/CVF International Conference on Computer Vision (ICCV).

[35] Kong L., Shen C., Yang J. FastFlowNet: A Lightweight Network for Fast Optical Flow Estimation. Proceedings of the 2021 IEEE International Conference on Robotics and Automation (ICRA).

[36] Lin T.-Y., Maire M., Belongie S., Hays J., Perona P., Ramanan D., Dollár P., Zitnick C.L., Fleet D., Pajdla T., Schiele B., Tuytelaars T. (2014). Microsoft COCO: Common Objects in Context. Proceedings of the Computer Vision—ECCV 2014.

[37] Xu H., Zhang J., Cai J., Rezatofighi H., Tao D. GMFlow: Learning Optical Flow via Global Matching. Proceedings of the IEEE/CVF Conference on Computer Vision and Pattern Recognition (CVPR).

[38] Wang C., Luo B., Zhang Y., Zhao Q., Yin L., Wang W., Su X., Wang Y., Li C. (2021). DymSLAM: 4D Dynamic Scene Reconstruction Based on Geometrical Motion Segmentation. IEEE Robot. Autom. Lett..

[39] Sturm J., Engelhard N., Endres F., Burgard W., Cremers D. A Benchmark for the Evaluation of RGB-D SLAM Systems. Proceedings of the 2012 IEEE/RSJ International Conference on Intelligent Robots and Systems.

[40] Liu Y., Miura J. (2021). RDMO-SLAM: Real-Time Visual SLAM for Dynamic Environments Using Semantic Label Prediction With Optical Flow. IEEE Access.

[41] Liu Y., Miura J. (2021). RDS-SLAM: Real-Time Dynamic SLAM Using Semantic Segmentation Methods. IEEE Access.

[42] Cheng S., Sun C., Zhang S., Zhang D. (2023). SG-SLAM: A Real-Time RGB-D Visual SLAM Toward Dynamic Scenes With Semantic and Geometric Information. IEEE Trans. Instrum. Meas..

[43] Palazzolo E., Behley J., Lottes P., Giguère P., Stachniss C. ReFusion: 3D Reconstruction in Dynamic Environments for RGB-D Cameras Exploiting Residuals. Proceedings of the 2019 IEEE/RSJ International Conference on Intelligent Robots and Systems (IROS).

[44] Handa A., Whelan T., McDonald J., Davison A.J. A Benchmark for RGB-D Visual Odometry, 3D Reconstruction and SLAM. Proceedings of the 2014 IEEE International Conference on Robotics and Automation (ICRA).

[45] Hui T.-W., Tang X., Loy C.C. LiteFlowNet: A Lightweight Convolutional Neural Network for Optical Flow Estimation. Proceedings of the IEEE Conference on Computer Vision and Pattern Recognition (CVPR).

